# Apoptosis on the move

**DOI:** 10.1007/s10495-018-1462-y

**Published:** 2018-06-01

**Authors:** Patrycja Nowak-Sliwinska, Arjan W. Griffioen

**Affiliations:** 1 School of Pharmaceutical Sciences, University of Geneva, University of Lausanne, Geneva, Switzerland; 2 Angiogenesis Laboratory, Department of Medical Oncology, VU University Medical Center, 1081 HV Amsterdam, The Netherlands

In the efforts to become a stronger and better recognized high quality journal for publication of research on the regulation of cell death, the new editors are dedicated to provide a yearly editorial on the best papers published in *Apoptosis*, called ‘*The editors’ choice*’. Furthermore, in the last editorial we have announced to changing the journal’s cover design on a yearly basis. For this we encouraged all authors to submit their microscopy images related to apoptotic cell death [1]. As the deadline has passed we have elected the winning cover image, being the one that is now featuring the cover of this issue, showing the apoptopodia of monocytic cells treated with ultraviolet light. A short presentation of three of the submitted images is given as well.

## The editors’ choice

Several excellent papers have been published in Apoptosis over the last years. Some of them are highlighted here. It was reported by Liu that pretreatment with a component of vine tea (*Ampelopsis grossedentata*), dihydromyricetin (DMY), can significantly protect against hypoxia–reoxygenation injury by suppression of apoptosis and necrosis. This effect correlated to the activity of PI3K/Akt and HIF-1 alpha signaling. It is suggested that DMY can be used for clinical treatment of acute myocardial infarction [2]. Another high impact study was the investigation of LPS mediated cardiomyocyte apoptosis, which is a mechanism of sepsis-induced cardiac cell death. It was found that LPS stimulation inhibited microRNA (miR)-499 resulting in upregulated expression of the miR-499 target genes SOX6 and PDCD4 and activation of the Bcl-2 family pathway [3]. In a study by Yu et al. the effect of melatonin in endoplasmatic reticulum stress was described [4]. In both in vitro and in vivo studies, melatonin was able to reduce cardiomyocyte ischemia–reperfusion injury. In the field of cancer research a paper was published on the combination treatment of glioblastoma with the natural flavonoids luteolin and silibinin. This drug combination inhibited the growth of glioblastoma cells in vitro more efficiently than conventional chemotherapy (e.g. temozolomide). The effect was mediated through induction of apoptosis and full blockade of invasion and migration, through suppression of protein kinase C, downregulation of iNOS and induction of miR-7-1-3p. The flavonoid combination also inhibited rapamycin-induced autophagy, a pathway that can cause survival of cells [5]. A study by Chang et al. showed that angiotensin II is able to induce apoptosis in endothelial cells, which could be suppressed by atorvastatin, the lipid-lowering drug Lipitor [6]. Finally, Raj found that anacardic acid can sensitize TRAIL resistant cancer cells for induction of apoptosis by upregulation of the death receptors DR4 and DR5. This result may be of impact for the development of TRAIL-based therapy that is currently in clinical studies [7]. Apoptosis also published a number of high-impact reviews that are worth mentioning. These papers were on the mitochondrial dynamics in maintaining cell integrity and preventing carcinogenesis [8], stem cell death and survival in heart regeneration and repair [9] and macroautophagy and mitochondrial ROS in cancer therapy [10].

## The cover image contest

**Fig. 1:**
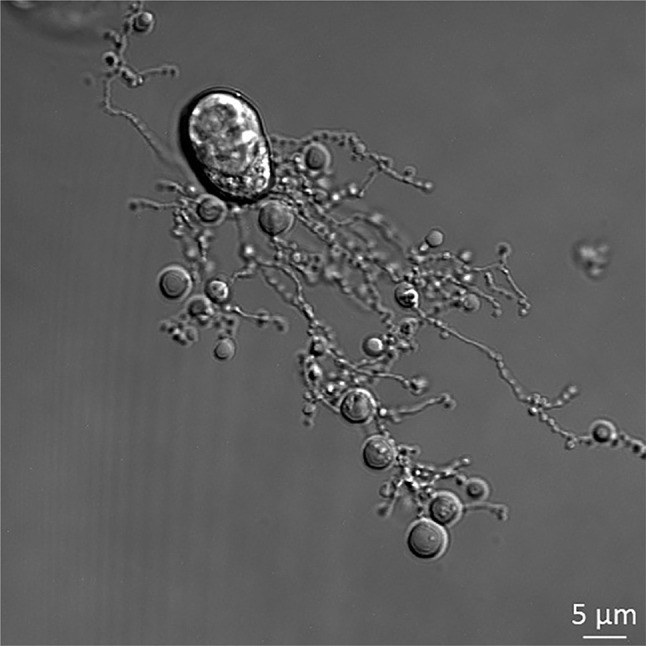
Human THP-1 monocytic cells treated with ultraviolet irradiation to induce apoptosis. During apoptosis, monocytes can generate ‘beads-on-a-string’ protrusions, known as beaded-apoptopodia, to mediate the formation of apoptotic bodies

With the *Apoptosis* editor change in 2017 it was planned to improve the appearance of the journal and make it more attractive to the readership. This will be done by a yearly change of the cover using images related to the process of apoptosis. The contest, which was started in 2017, was won by Dr. Ivan Poon (VIC, Australia), who submitted an image that is on the cover of this issue. It shows a cell of the human THP-1 monocytic cell line, treated with ultraviolet irradiation to induce apoptosis. During apoptosis, monocytes can generate ‘beads-on-a-string’ protrusions, known as beaded-apoptopodia, to mediate the formation of apoptotic bodies. Apoptosis is a process that occurs in essentially all tissues as part of normal development and pathogenic processes including chronic inflammation and infection [11, 12]. During apoptosis, dying cells can disassemble into smaller membrane-bound extracellular vesicles called apoptotic bodies (ApoBDs). Under certain pathological conditions, ApoBDs can carry cellular contents including microRNA and cytokines to regulate tissue repair and inflammation. Therefore, the formation of ApoBDs is an important process downstream of apoptotic cell death that could regulate intercellular communication [13]. For many years, ApoBD formation was thought to be an unregulated process that occurs stochastically at later stages of cell death. However, Dr Poon’s team has challenged this paradigm and recently demonstrated that the formation of ApoBD is a highly regulated process controlled by distinct morphological steps [14, 15]. In particular, Dr Poon’s group discovered the unique ability of monocytes to generate ApoBD via the formation of a novel ‘beads-on-a-string’ membrane protrusions called beaded-apoptopodia [14] (Fig. 1). Understanding the role of this process in disease settings may lead to development of new therapeutics that may target the apoptotic cell disassembly process.

**Fig. 2:**
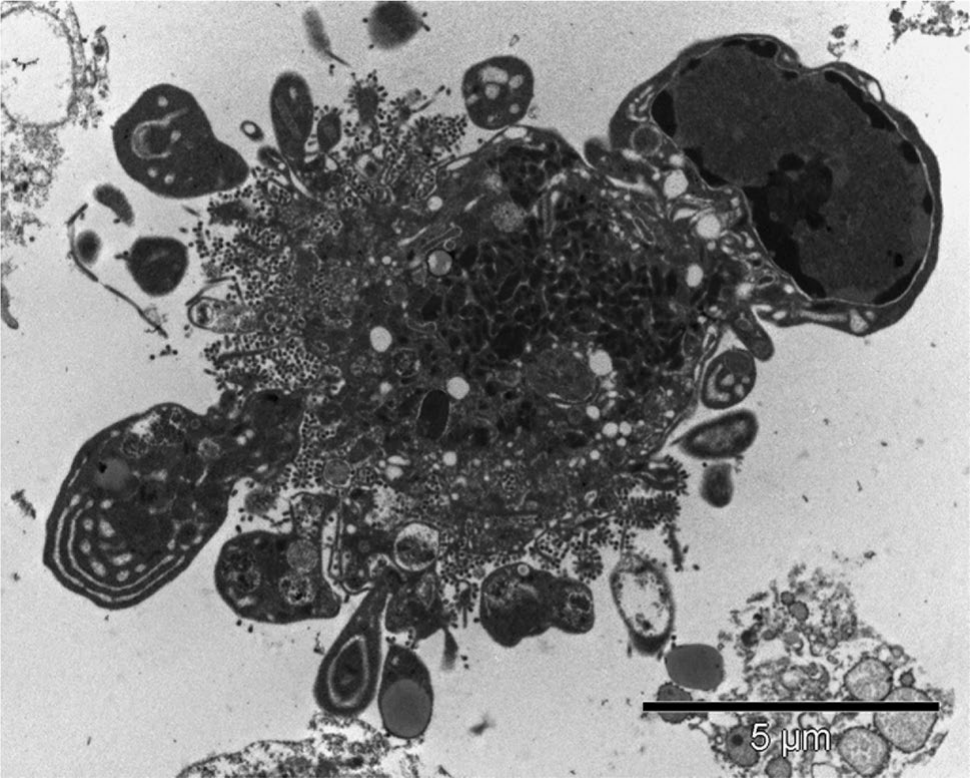
Dog kidney MDCK cells (widespread use in viral infections studies) infected with influenza virus strain WSN and treated with caspase inhibitor z-Vad. Of note: cell surface blebs can be seen, containing cell organelles; immature viral particles are visible in the cell cytoplasm, whereas mature viral particles budding from cell membrane can be observed; condensed and marginalized chromatin inside the cell nucleus can be seen (upper right)

Several other stunning images have been submitted. Three of them deserve a short introduction. An electron microscopy image submitted by dr. Walter Malorni and Antonella Tinari (Rome, Italy) shows an apoptotic MDCK dog kidney cell infected with influenza virus strain WSN and treated with caspase inhibitor z-Vad. Influenza A virus infection leads to cell death by apoptosis. However, elevated lysosomal activity and an abundance of autophagosomes can be observed when apoptosis is inhibited. Hence, the balance between apoptotic mechanisms and autophagic cytoprotection pathway is essential for successful viral spreading or its abortive replication. On the cell side, apoptotic cell death or autophagic cell survival can in turn be pivotal in the pathogenetic mechanisms of infection. Very clearly visible are the cell surface blebbing, viral budding from the cell membrane and chromatin marginalization (Fig. 2) [16–18].

**Fig. 3:**
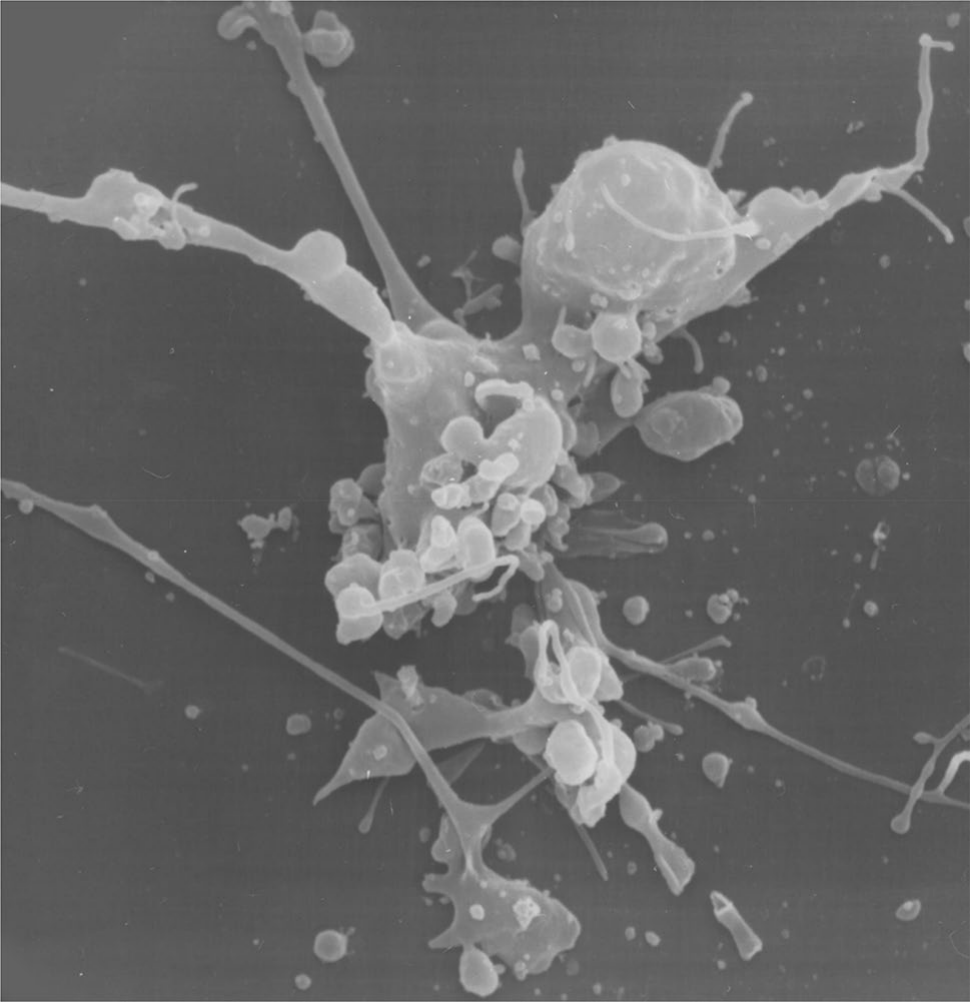
Scanning electron micrograph of SKBr-3 breast cancer cells undergoing apoptosis after treatment with 3 µM dihydroindolizino[7,6,5-kl]-acridinium chloride for 12 h

Dr. Stanslas (Serdang, Selangor, Malaysia) provided a scanning electron microscopy image of metastatic mammary carcinoma SKBr-3 cells that were treated with an anti-cancer drug (Fig. 3). The drug was developed after synthesizing tetra- and penta-cyclic DNA interactive acridines in an effort to discover potential anticancer agents [19]. One of the lead compounds, dihydroindolizino[7,6,5-kl]-acridinium chloride (DS, Fig. 1) displayed growth inhibitory activity at sub-micromolar concentration range in panels of breast and non-small cell lung cancer cell lines [20]. The compound formed a binding ‘hot spot’ in DNA with the planar pyridoacridine moiety intercalating at G–C sequences and the pyrrolidinium fragment occupying minor or major grooves [21]. Elucidation of its mechanism of action revealed topoisomerase II as a target, with selectivity for the α isoform over the β form [4]. Unlike the anticancer drug amsacrine (Fig. 1), which is also an acridine derivative, DS was not susceptible to *P*-glycoprotein-mediated drug efflux and retained activity in lung and breast cancer cells made resistant to the topoisomerase II inhibitors (etoposide and doxorubicin) [20]. In addition, the compound was shown to be more potent than amsacrine and etoposide in breast cancer cells with varying molecular characteristics [20]. Assessment of mode of cell death induced by the compound revealed SKBr-3 breast cancer cells underwent apoptosis, as observed with the aid of scanning electron microscopy (Fig. 3).

With the selection of the new image to the cover, we hope that all authors will get inspired to share with us their most striking images for future covers of *Apoptosis*.
